# 5-Methoxy-2-aminoindane Reverses Diet-Induced Obesity and Improves Metabolic Parameters in Mice: A Potential New Class of Antiobesity Therapeutics

**DOI:** 10.1021/acsptsci.4c00353

**Published:** 2024-07-30

**Authors:** Saja Baraghithy, Asaad Gammal, Anna Permyakova, Sharleen Hamad, Radka Kočvarová, Yael Calles, Joseph Tam

**Affiliations:** Obesity and Metabolism Laboratory, The Institute for Drug Research, School of Pharmacy, Faculty of Medicine, The Hebrew University of Jerusalem, Jerusalem 9112001, Israel

**Keywords:** psychedelic therapy, obesity, insulin resistance, metabolic syndrome, food addiction, hepatic steatosis

## Abstract

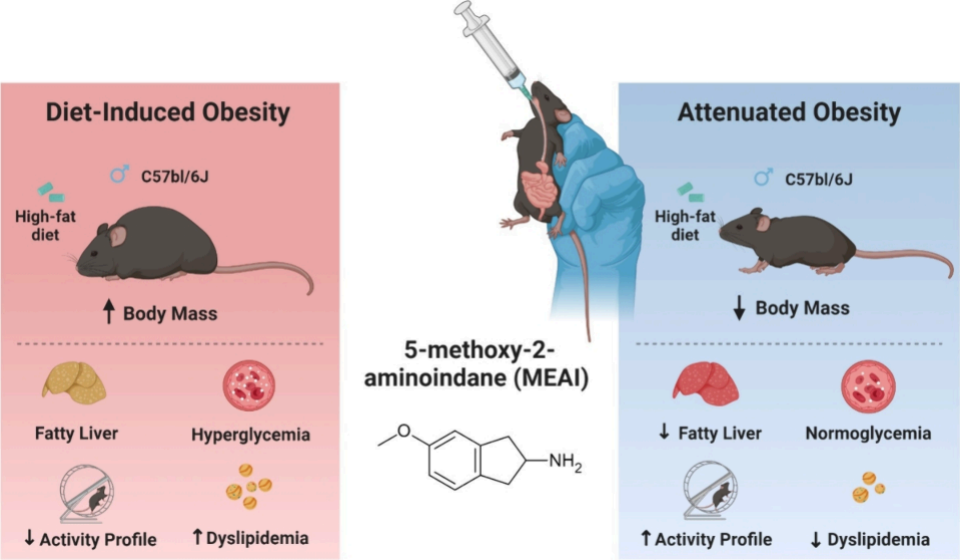

The escalating prevalence of obesity and its related disorders represents a daunting global health challenge. Unfortunately, current pharmacological interventions for obesity remain limited and are often associated with debilitating side effects. Against this backdrop, the psychoactive aminoindane derivative 5-methoxy-2-aminoindane (MEAI) has gained considerable attention for its ability to induce a pleasurable, alcohol-like sensation while curbing alcohol consumption. Given the potential impact of MEAI on food addiction and energy homeostasis, we examined its metabolic efficacy on appetite regulation, obesity, and related comorbidities under acute and chronic settings, utilizing a mouse model of diet-induced obesity (DIO). Our results demonstrated that MEAI treatment significantly reduced DIO-induced overweight and adiposity by preserving lean mass and decreasing fat mass. Additionally, MEAI treatment exhibited positive effects on glycemic control by attenuating DIO-induced hyperglycemia, glucose intolerance, and hyperinsulinemia. Furthermore, MEAI reduced DIO-induced hepatic steatosis by decreasing hepatic lipid accumulation and lowering liver triglyceride and cholesterol levels, primarily by inhibiting *de novo* lipid synthesis. Metabolic phenotyping revealed that MEAI increased energy expenditure and fat utilization while maintaining food consumption similar to that of the vehicle-treated group. Lastly, MEAI normalized voluntary locomotion actions without any overstimulatory effects. These findings provide compelling evidence for the antiobesity effects of MEAI treatment and call for further preclinical testing. In conclusion, our study highlights the potential of MEAI as a novel therapeutic approach for treating obesity and its associated metabolic disorders, offering hope for the development of new treatment options for this global health challenge.

The prevalence of obesity has reached epidemic proportions, with approximately one billion people worldwide projected to be affected by 2030, including approximately one in five women and one in seven men, according to the recently published World Obesity Atlas 2022 by the World Obesity Federation.^1^ Obesity is a chronic disease that has been linked to several conditions, including cardiovascular disease (CVD), type 2 diabetes (T2D), and metabolic dysfunction-associated steatotic liver disease (MASLD).^2^ While multiple metabolic factors have been associated with the development of obesity, the underlying molecular mechanisms are not yet fully understood. Despite the gravity of the situation, the lack of effective antiobesity treatments poses a significant challenge.

Several factors, including eating habits, fast food consumption, personality traits, depression, and genetics, have been implicated in the etiologies of obesity. Recently, the notion of food addiction has gained attention as a potential explanation for the obesity epidemic, with links observed between food addiction and substance-related and eating disorders (most prominently binge eating).^3^ The similarities between food addiction and drug dependence, including an overactive response in the mesolimbic reward circuits, contribute to an inability to control food intake and maintain weight loss, leading to a cycle of binge eating that is difficult to break.^4,5^ This pattern is similar to what is seen in drug addiction, where the activation of these reward circuits contributes to an inability to control drug use.^6−8^ The similarities between food addiction and drug dependence suggest that similar therapeutic approaches may be effective in treating both obesity and addictive disorders.

Psychedelics, including lysergic acid diethylamide (LSD), mescaline, psilocybin, and *N*,*N*-dimethyltryptamine (DMT), interact with the serotonin receptors, particularly 5-hydroxytryptamine (5-HT)-2C, 5-HT2A, and 5-HT1A.^9,10^ These receptors are densely located in the prefrontal cortex and mesolimbic dopamine pathways, areas involved in emotion control, reward, and food intake.^11^ Previous studies have shown that stimulation of the serotonergic system induces weight reduction and decreases food intake.^12^ Moreover, cerebral 5-HT2A binding was significantly and positively correlated with BMI and provided a predictive value for weight loss after gastric bypass surgery.^13,14^ Importantly, psychedelics may offer a unique approach to addressing both addiction and obesity by disrupting rigid behavioral patterns, promoting cognitive flexibility, and increasing neuronal plasticity.^15^

5-Methoxy-2-aminoindane (MEAI) is a psychoactive compound that belongs to the aminoindane class and has gained popularity among recreational users due to its reported ability to produce mild euphoric effects similar to those of alcohol. Importantly, MEAI also dampens the desire to consume alcoholic beverages, thus reducing binge-drinking behavior.^16,17^ Given these observations, we evaluated the potential impact of MEAI on food addiction behavior, including its ability to regulate appetite and weight gain. Our study found that both acute and chronic MEAI administrations hold the potential to modulate energy balance and metabolism. The effects of repeated administration of MEAI, at a dose of 40 mg/kg/day, were evaluated in a diet-induced obese (DIO) mouse model, where it was found to substantially mitigate weight gain and adiposity while maintaining lean mass and reducing overall fat mass. MEAI administration also alleviated DIO-induced hyperglycemia, glucose intolerance, and hyperinsulinemia, highlighting its therapeutic potential in regulating glucose metabolism. Furthermore, MEAI ameliorated DIO-induced hepatic steatosis, evident from reduced hepatic lipid accumulation along with lowered liver triglyceride and cholesterol levels. Our findings suggest that MEAI treatment has the potential to modulate metabolism and counteract obesity, highlighting the ability of psychedelics and their related ligands to treat metabolic syndrome.

## Materials and Methods

### Materials

2,3-Dihydro-5-methoxy-1*H*-inden-2-amine hydrochloride (MEAI) (96% purity), synthesized by Vanamali Organics Pvt., Ltd., was provided by Clearmind Medicine Inc.

### Animals

The experimental protocol employed in this study was approved by the Institutional Animal Care and Use Committee of the Hebrew University, which is an AAALAC International accredited institute (Ethic approval number MD-21-16798). Animal studies were conducted in compliance with the ARRIVE guidelines,^18^ which aim to improve the transparency and reproducibility of preclinical research. The principle of replacement, refinement, or reduction was followed to minimize the number of animals used in this study. All of the animals were housed in specific pathogen-free (SPF) conditions, with no more than five animals of the same gender and dose group per cage, in standard plastic cages, with natural soft sawdust provided as bedding.

### Acute Effect of MEAI on Food Intake and Activity

Male 6-week-old C57Bl/6 mice (Harlan, Israel) were maintained in standard conditions under a 12 h light/dark cycle and fed *ad libitum*. A total of 32 animals were divided into four experimental groups (*N* = 8 mice per group) receiving single doses of 40, 60, or 100 mg/kg of MEAI or vehicle (sterile water), which was administered via oral gavage 2 h prior to the dark phase. The animals were monitored for 48 h postdose for drug tolerability, food and water intakes, and activity and metabolic parameters. At the end of the experiment, the animals were euthanized, and tissues (brain, liver, fat, and kidney) and blood were collected and stored in a frozen state for future analyses.

### Effect of MEAI on Obesity

Male C57BL/6 mice were used to establish DIO by feeding them a high-fat diet (HFD; 60% cal/L fat, 20% cal/L protein, and 20% cal/L carbohydrates; Research Diet, D12492) for 18 weeks. After this period, mice were treated with either vehicle (sterile water, *N* = 8) or MEAI (*N* = 11) daily for 28 days by gavage at a dose of 40 mg/kg/day. Age-matched control mice (*N* = 10) on a standard diet (STD; 14% kcal fat, 24% kcal protein, 62% kcal carbohydrates; NIH-31 rodent diet) received vehicle daily. The body weight of all mice was monitored daily, and total body fat and lean mass were determined by EchoMRI-100H (Echo Medical Systems LLC, Houston, Texas, USA). On day 29, at the end of the experimental period, mice were euthanized by a cervical dislocation under anesthesia. The kidneys, brain, liver, and fat pads were removed and weighed, and samples were either snap-frozen or fixed in buffered 4% formalin. Trunk blood was collected to determine the biochemical parameters.

### Sucrose Preference Test

Thirteen-week-old male C57BL/6 mice, maintained on a STD and housed individually, were habituated to two water bottles in their home cage for 48 h prior to the test. Baseline intake was measured by weighing the bottles. On the test day (day 1), 2 h before the onset of the dark phase, fresh water and a 1.5% sucrose solution were added to the bottles and mice were subsequently treated orally with MEAI (40 mg/kg, *N* = 8) or sterile water (*N* = 8). The mice were allowed to freely drink from either bottle for 24 h, after which the bottles were weighed to measure consumption. The study was repeated for an additional day (day 2), with the bottles switched in position (in the cage) to account for side preference. The sucrose and water intake over the 2 days were averaged, and the sucrose preference index was calculated as the average consumed sucrose solution divided by the average volume of total consumed liquid (average water plus average sucrose solution).

### Multiparameter Metabolic Assessment

The metabolic profiles and food and water intakes of the mice were assessed using the Promethion High-Definition Behavioral Phenotyping System (Sable Instruments, Inc., Las Vegas, Nevada, USA). MetaScreen software version 2.2.18.0 was used for data acquisition and instrument control. Raw data were processed using ExpeData version 1.8.4 with an analysis script detailing all aspects of the data transformation. Mice with free access to food and water, housed at temperatures of (22–23 °C), were subjected to a standard 12 h light/12 h dark cycle, which consisted of a 48 h acclimation period followed by 24 h of sampling after dosing. Respiratory gases were measured using the GA-3 gas analyzer (Sable Systems, Inc., Las Vegas, Nevada, USA) employing a pull-mode negative-pressure system. Airflow was measured and controlled by an FR-8 instrument (Sable Systems, Inc., Las Vegas, Nevada, USA) at a flow rate of 2000 mL/min. Water vapor was continuously measured, and its dilution effect on O_2_ and CO_2_ was mathematically compensated. Respiratory exchange rate (RER) was calculated as the ratio of CO_2_ produced (VCO_2_) to O_2_ consumed (VO_2_) using eq 1):
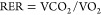
1

Total energy expenditure (TEE) was calculated using VO_2_ and RER, according to eq 2:
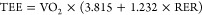
2

Fat oxidation (FO) and carbohydrate oxidation (CHO) were calculated using VO_2_ and VCO_2_ based on eqs 3 and 4, respectively:
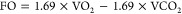
3
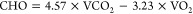
4

Energy balance and energy flux were derived from eqs 5 and 6:
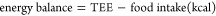
5
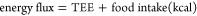
6

The thermic effect of food for each animal was calculated based on its individual food intake in a period of 24 h according to the specific percentage of carbohydrates, fats, and proteins in the consumed diet.

The basal energy expenditure was calculated based on the mean energy expenditure (EE) during the lowest EE in a 30 min period, in kilocalories per hour (kcal/h). This calculation represented the animal’s basal metabolic rate.

The activity energy expenditure was calculated based on eq 7:
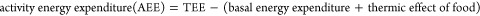
7

### Wheel Running and Locomotor Activity

The assessment of wheel running and locomotor activity was performed by using the Promethion High-Definition Behavioral Phenotyping System (Sable Instruments, Inc., Las Vegas, Nevada, USA). Wheel revolutions were measured with a monitor that recorded voluntary wheel running activity, and locomotor activity was quantified using disruptions of infrared XYZ beam arrays with a beam spacing of 0.25 cm. Pedestrian locomotion represented the sum of all directed ambulatory locomotion within the beam break system, using a speed cutoff of 1 cm/s. Total distance represented the sum of all distances traveled within the beam break system, not including any distance run on the wheel in meters (m); this included fine movements (such as grooming and scratching).

### Glucose Tolerance Test (ipGTT) and Insulin Tolerance Test (ipITT)

On day 25 of the experiment, mice were subjected to an overnight fasting and then injected with glucose (1.5 g/kg i.p.) on the following day (day 26). Blood glucose levels were determined at 0, 15, 30, 45, 60, 90, and 120 min after injection using the Contour glucometer (Bayer, Pittsburgh, Pennsylvania, USA). The mice were then fasted for 6 h the next day (day 27) before being administered insulin (0.75 U/kg, i.p.; Actrapid vials, Novo Nordisk A/S, Bagsværd, Denmark). Blood glucose levels were determined at the same intervals as those described above. To assess insulin resistance, the homeostasis model assessment insulin resistance (HOMA-IR) was calculated as fasting serum insulin ([μU/mL] × fasting plasma glucose [mmol/L]/22.5). The relative insulin sensitivity index (ISI) was calculated as 1/(glucose × insulin) × 1000, with glucose expressed as mg/dL and insulin as mU/L.

### Blood Biochemistry

Serum levels of alanine aminotransferase (ALT), aspartate aminotransferase (AST), alkaline phosphatase (ALP), cholesterol, triglycerides, high-density lipoprotein (HDL), and low-density lipoprotein (LDL) were quantified post-termination using the Cobas C-111 chemistry analyzer (Roche, Switzerland). Fasting blood glucose was measured using the Contour glucometer (Bayer, Pittsburgh, Pennsylvania, USA), while serum insulin was determined using an Ultra-Sensitive Mouse Insulin ELISA kit (Cat# 90080, Crystal Chem, Inc., Elk Grove Village, Illinois, USA). Serum-free fatty acid content was determined using a Free Fatty Acid Assay Kit (Cat# ab65341, Abcam, Cambridge, UK). Serum leptin (Cat# EZML-82K, Millipore Sigma, Burlington, Massachusetts, USA), serotonin (Cat# ab133053, Abcam, Cambridge, UK), and adpidonectin (Cat# 80569, Crystal Chem, Inc., Elk Grove Village, Illinois, USA) levels were quantified by ELISAs.

### Hepatic Triglyceride and Cholesterol Contents

At the time of animal sacrifice, liver tissue was extracted following the established protocol described previously.^19^ The extracted liver tissue was then analyzed for cholesterol and triglyceride contents using a Cobas C-111 chemistry analyzer (Roche, Switzerland).

### Histopathology

Paraffin-embedded liver sections (5 μm) from five animals per group were processed for hematoxylin–eosin staining. Liver images were captured using a Zeiss Axio Scope A1 light microscope (Carl Zeiss AG, Jena, Germany) equipped with a Zeiss AxioCam ICc 5 color camera. Ten random 40× fields of view were taken from each animal to obtain representative images.

### Oil Red O Staining

Liver cryosections (8 μm) were stained with Oil Red O (Cat# ab150678; Abcam, Cambridge, UK) following the manufacturer’s protocol. Images were acquired as described above. For quantitative analysis of Oil Red O staining, the area of lipid droplets in the liver cryosections was measured using ImageJ software.

### Ligand Binding Assays

MEAI at 10 μM was tested in ligand binding assays at Eurofins Inc., as described previously.^20^ Compound binding was calculated as a % inhibition of the binding of a ligand specific for each target. Results showing an inhibition or stimulation higher than 50% are considered to represent significant effects of MEAI. In each experiment, and if applicable, the respective reference compound was tested concurrently with MEAI and the data were compared with historical values determined at Eurofins. The experiment was accepted in accordance with the Eurofins validation Standard Operating Procedure.

### 5-HT2B–Calcium Influx Assay

This assay was performed using the screening services of Eurofins. Evaluation of the agonistic activity of MEAI at the human 5-HT2B receptor expressed in BA/F3 cells was determined by measuring its effect on cytosolic Ca^2+^ ion mobilization using a fluorimetric detection method. The cells were suspended in HBSS buffer (Invitrogen) complemented with 20 mM Hepes and then distributed in microplates at a density of 5 × 104 cells/well. The fluorescent probe (Fluo-8, AAT Bioquest, San Francisco, California) mixed with probenicid in HBSS buffer (Invitrogen) complemented with 20 mM Hepes (Millipore, Burlington, Massachusetts) (pH 7.4) was then added into each well and equilibrated with the cells for 60 min at 30 °C. Thereafter, the assay plates were positioned in a microplate reader (FLIPR Tetra, Molecular Devices, San Jose, California), which was used for the addition of the test compound, reference agonist, or HBSS buffer (basal control) and for the measurements of changes in fluorescence intensity that varies proportionally to the free cytosolic Ca^2+^ ion concentration. For stimulated control measurements, serotonin at 0.25 μM was added in separate assay wells. The results were expressed as a percent of the control response to serotonin at 0.25 μM. The standard reference agonist was serotonin, which was tested in each experiment at several concentrations to generate a concentration–response curve from which its EC50 value was calculated.

### Western Blotting

Liver samples were prepared in a RIPA buffer (25 mM Tris–HCl pH 7.6, 150 mM NaCl, 1% NP-40, 1% sodium deoxycholate, 0.1% SDS), and the homogenates were prepared by using the Bullet Blender and zirconium oxide beads (catalog no. ZROB10, Next Advanced, Inc., New York, USA). Protein concentrations were measured with the Pierce BCA Protein Assay Kit (catalog no. 23225, Thermo Scientific, Illinois, USA). Samples were resolved by SDS-PAGE (4–15% acrylamide, 150 V) and transferred to PVDF or nitrocellulose membranes using the Trans-Blot Turbo Transfer System (Bio-Rad, California). The membranes were then incubated for 1 h in 5% milk (in 1× M TBS-T) to block unspecific binding. The membranes were incubated overnight with cluster of differentiation 36 (CD36; Abcam, Cambridge, UK; Cat# ab252922), stearoyl-CoA desaturase 1 (SCD1; Cell Signaling Technology, Danvers, Massachusetts; Cat# 2794S), fatty acid synthase (FASN; Cell Signaling Technology, Danvers, Massachusetts, USA; Cat# 3180S), 5′-adenosine monophosphate (AMP)-activated protein kinase alpha (AMPKα; Cell Signaling Technology, Danvers, Massachusetts, USA; Cat# 2532S), phosphorylated-AMPKα (Cell Signaling Technology, Danvers, Massachusetts, USA; Cat# 2535S), acetyl-CoA carboxylase (ACC; Cell Signaling Technology, Danvers, Massachusetts, USA; Cat# 3662S), and phosphorylated ACC (Cell Signaling Technology, Danvers, Massachusetts, USA; Cat# 3661S) antibodies at 4 °C. Antirabbit/mouse horseradish peroxidase (HRP)-conjugated secondary antibodies were used for 1 h at room temperature, followed by chemiluminescence detection using Clarity Western ECL Blotting Substrate (Bio-Rad, California). Densitometry was quantified by using ImageJ software. Quantification was normalized to anti-β actin antibody (Abcam, Cambridge, UK; Cat# ab49900) or valosin-containing protein (VCP) (Abcam, Cambridge, UK; Cat# ab204290).

### Real-Time PCR

mRNA from livers was extracted using a Bio-Tri RNA lysis buffer (Bio-Lab, Israel), followed by DNase I treatment (Thermo Scientific, Illinois, USA), and reverse transcribed using the qScript cDNA Synthesis kit (Quantabio, Beverly, Massachusetts, USA). Real-time PCR was performed using an iTaq Universal SYBR Green Supermix (Bio-Rad, California, USA) and the CFX connect ST system (Bio-Rad, California, USA). The full list of primers is available as Table S1.

### Statistics

The data are presented as mean ± SEM. Statistical analysis was conducted using GraphPad Prism 9.0 software (GraphPad Software, California, USA). Differences between the two groups were determined using an unpaired two-tailed Student’s *t*-test. For comparisons involving multiple groups and time-dependent variables, ANOVA was employed, followed by Tukey’s multiple comparisons test. Statistical significance was considered when *p*-values were less than 0.05. The EE ANCOVA analysis done for this work was provided by the NIDDK Mouse Metabolic Phenotyping Centers (MMPC) using their Energy Expenditure Analysis page^21^ and supported by grants DK076169 and DK115255 (Table S2).

## Results

### Acute Response of MEAI Administration on Energy Balance and Activity

**Figure 1:**
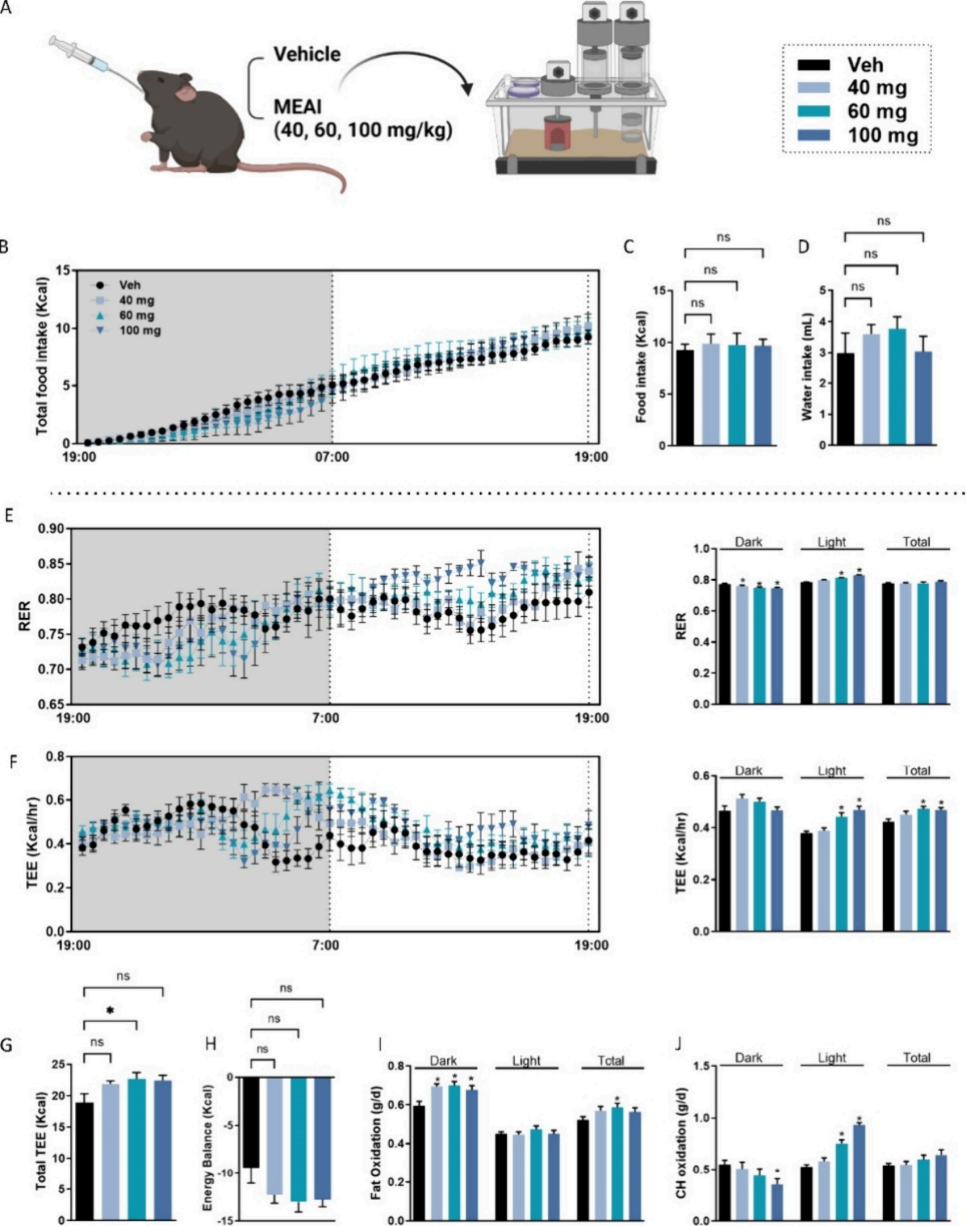
Effects of acute MEAI administration on food intake patterns and energy utilization. Experimental design (created with BioRender.com) (A), cumulative food intake (B), sum of food intake (C), sum of water intake (D), respiratory exchange rate (RER) (E), average energy expenditure (TEE/h) (F), total energy expenditure (TEE) (G), energy balance (kcal) (H), fat oxidation (I), and carbohydrate oxidation (J). Data represent the mean ± SEM from six to eight mice per group. *P < 0.05 relative to the vehicle-treated group.

To assess the immediate effects of MEAI on food intake patterns and respirometric parameters, a single dose of 40, 60, or 100 mg/kg was administered 2 h prior to the onset of the dark phase, as depicted in Figure 1A. The results indicated that the drug was well-tolerated, with no observable changes in behavior at the 40 and 60 mg/kg dosages. However, two subjects in the 100 mg/kg group died within a few hours of the drug administration, suggesting reduced tolerance at this dose, which was exacerbated by metabolic testing stress in combination with the stress caused by metabolic testing, and thus they were excluded from the analysis. Minor changes in feeding patterns were observed during the active (dark) and inactive (light) phases following MEAI administration, but these changes did not reach statistical significance (Figure 1B,C). Moreover, there were no notable changes in water consumption (Figure 1D). In contrast to food intake, MEAI administration significantly impacted metabolic parameters. Doses of 60 and 100 mg/kg markedly increased the respiratory exchange ratio (RER) during the light phase (Figure 1E), indicating a shift toward carbohydrate utilization. This finding was aligned with the observed increases in both oxygen consumption (VO_2_) and carbon dioxide production (VCO_2_) (Figure S1A,B). Importantly, across the 24 h examined, a significant dose-dependent increase in hourly average energy expenditure levels (TEE/h) was observed, culminating in a significant elevation in total daily TEE, particularly at the 60 mg/kg dose (Figure 1F,G). Acute MEAI administration did not lead to immediate changes in the energy balance (Figure 1H). Furthermore, elevations in FO and CHO rates (Figure 1I,J) were evident, suggesting a shift in the body’s fuel preference toward both carbohydrates and fats at higher doses. Overall, these findings demonstrate that MEAI has the potential to acutely increase energy expenditure and promote changes in substrate utilization in mice.

**Figure 2:**
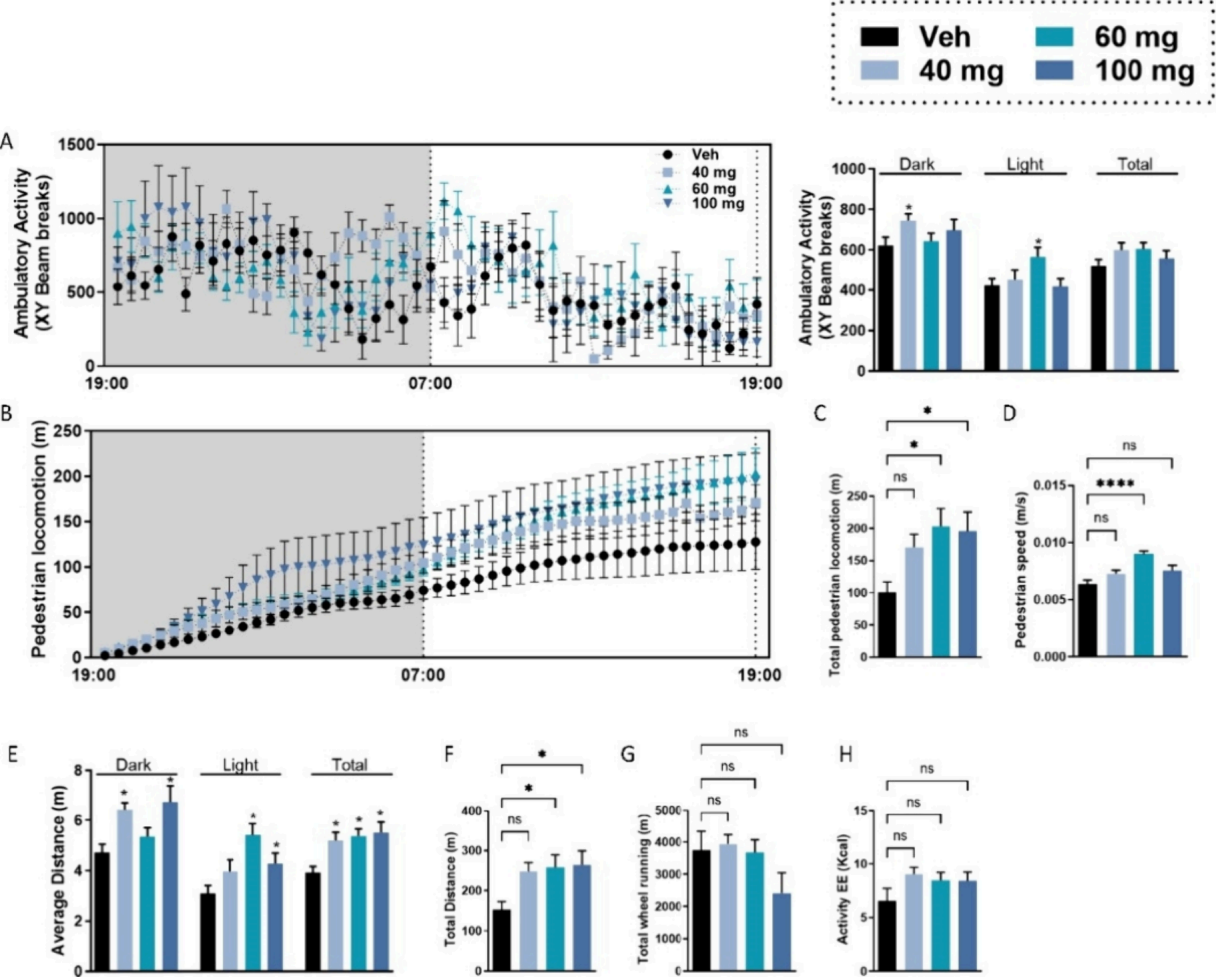
Alterations in the activity profile following acute MEAI administration. Total ambulatory activity (XY beam breaks) (A), cumulative pedestrian locomotion (B), sum of pedestrian locomotion (C), locomotion speed (D), average distance traveled (E), sum of total distance (F), wheel running (G), and activity-related energy expenditure (H). Data represent the mean ± SEM from six to eight mice per group. *P < 0.05 relative to the vehicle-treated group.

Building upon the observed changes in energy expenditure, we next examined the impact of MEAI on activity patterns in mice. While the total number of beam breaks, encompassing both ambulation and fine movements, exhibited minor alterations (Figure 2A), MEAI administration triggered a significant and dose-dependent increase in directed voluntary activity (Figure 2B–D). This targeted activity, encompassing movements associated with feeding, drinking, and grooming, showed a clear rise at doses of 40 mg/kg and above. Notably, the 60 mg/kg dose produced a significant increase in the pedestrian speed (Figure 2D). Furthermore, the average and total distance traveled within the cage displayed significant elevations across all doses (Figure 2E,F), suggesting an overall increase in movement. Interestingly, MEAI did not significantly affect the sum of the voluntary wheel running activity at any dose tested (Figure 2G). Finally, a modest insignificant rise in activity-specific energy expenditure was observed following MEAI administration (Figure 2H). These findings further support the potential of MEAI to impact energy balance via modulating activity patterns.

### Effect of MEAI on Sucrose Preference

**Figure 3:**
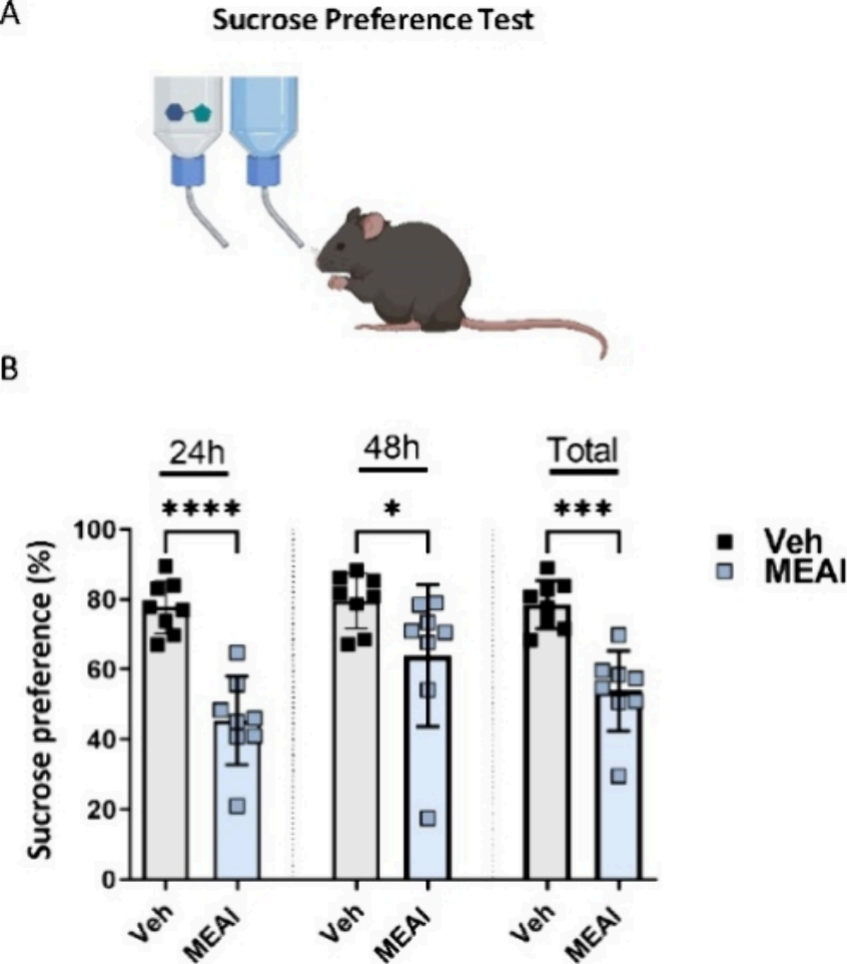
MEAI reduces sucrose preference. Two-bottle paradigm experimental design of the sucrose preference test (created with BioRender.com) (A) and percentage of sucrose preference over a the test period of 48 h (B). Data represent mean ± SEM from eight mice per group. *P < 0.05 relative to the vehicle-treated group.

To assess the impact of MEAI on sweet taste preference, we employed the sucrose preference test (SPT), a commonly used reward-based test to detect anhedonia (Figure 3A). Following a single injection, MEAI at a dosage of 40 mg/kg significantly reduced the acute preference of mice for sucrose solution without any accompanying decrease in water intake levels. This effect was most evident during the initial 24 h period, with a slight reduction noted during the subsequent 24 h period (Figure 3B). These results indicate that MEAI has the potential to impede reward stimuli, leading to decreased hedonic effects that are typically associated with palatable food.

### MEAI Ameliorates HFD-induced Obesity

**Figure 4:**
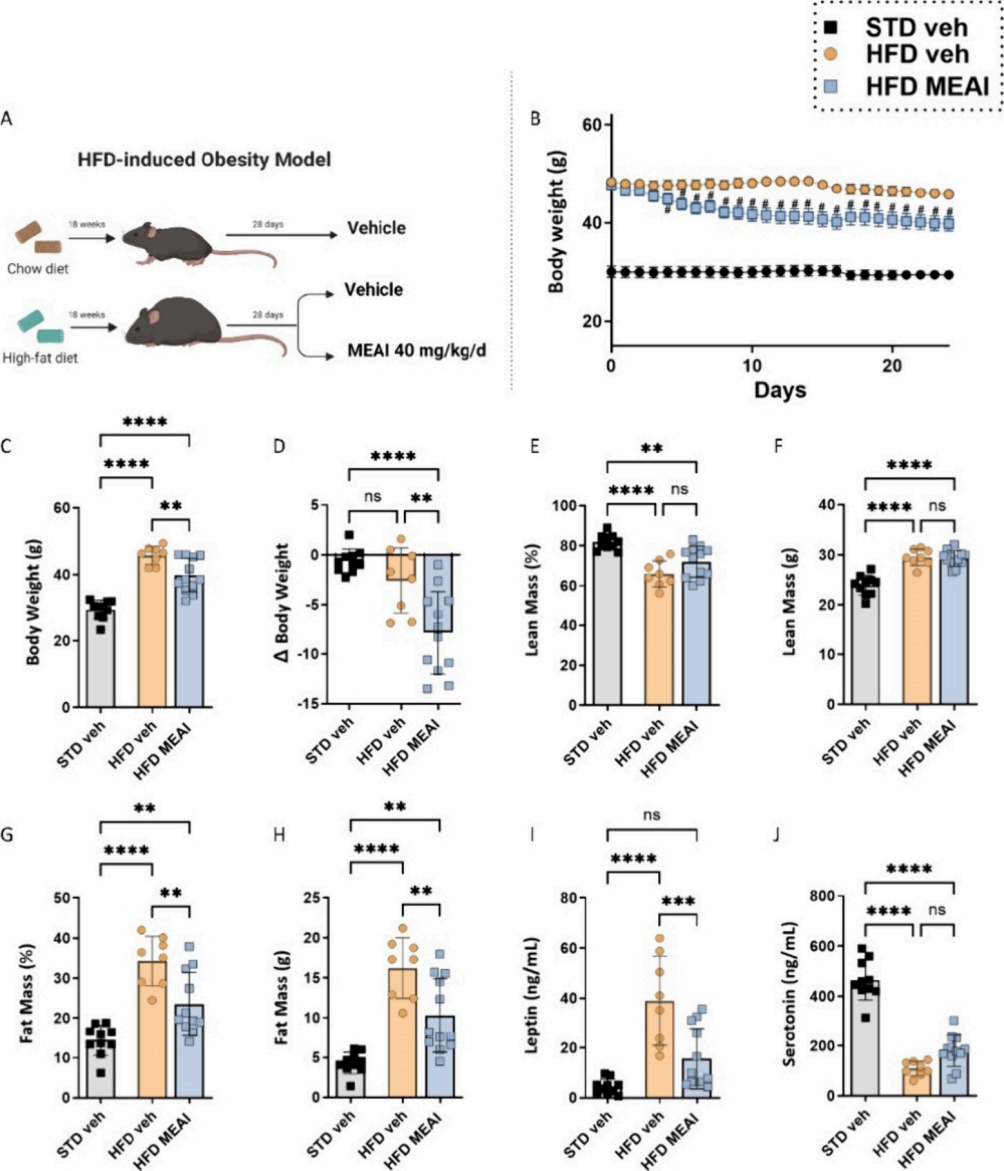
MEAI attenuates weight gain and body composition changes associated with obesity. Experimental design to test the efficacy of MEAI (40 mg/kg/day) in an HFD-induced obesity model (created with BioRender.com) (A), time-course change of body weight (B), body weight at the end of experiment (C), total body weight change at the end of experiment (D), lean mass percentage of overall body weight (E), lean mass in grams (F), fat mass percentage of overall body weight (G), fat mass in grams (H), serum leptin levels (I), and serotonin levels (J). Data represent mean ± SEM from 8–11 mice per group. *P < 0.05 relative to STD vehicle; #*P* < 0.05 relative to HFD vehicle.

Previous research has indicated that MEAI can decrease the desire to consume alcoholic beverages, potentially reducing binge-drinking behavior.^17^ Given these findings, we next investigated the impact of MEAI on food addiction behavior by assessing its metabolic efficacy in regulating appetite, in treating obesity, and its related abnormalities in a DIO mouse model (Figure 4A). To ensure a balance between efficacy and minimizing potential side effects, a suboptimal dose of 40 mg/kg/day of MEAI was employed for chronic exposure.

At baseline prior to drug treatment, the mice fed with an HFD exhibited significantly greater weight compared to the control group fed with STD. Over the 28-day treatment period, MEAI administration significantly reduced the body weight of HFD-fed mice (Figure 4B), resulting in an approximate 15% decrease in total body weight compared to the obese vehicle-treated group (Figure 4C,D). Additionally, MEAI treatment reduced adiposity associated with obesity in the DIO model, maintaining the lean body mass ratio and net lean mass (Figure 4E,F) while simultaneously reducing the overall fat mass (Figure 4G,H). Next, the effect of MEAI treatment on key metabolic hormones was evaluated. Notably, MEAI administration effectively countered the HFD-induced elevation of circulating leptin levels (Figure 4I). This suggests that MEAI may mitigate hyperleptinemia-induced leptin resistance, promoting feelings of fullness. In contrast, MEAI treatment did not significantly alter serum serotonin levels (Figure 4J), both the HFD vehicle- and MEAI-treated groups displayed lower serotonin levels compared to lean mice on a STD while a trend toward elevation in serotonin levels was observed in the MEAI-treated group.

**Figure 5:**
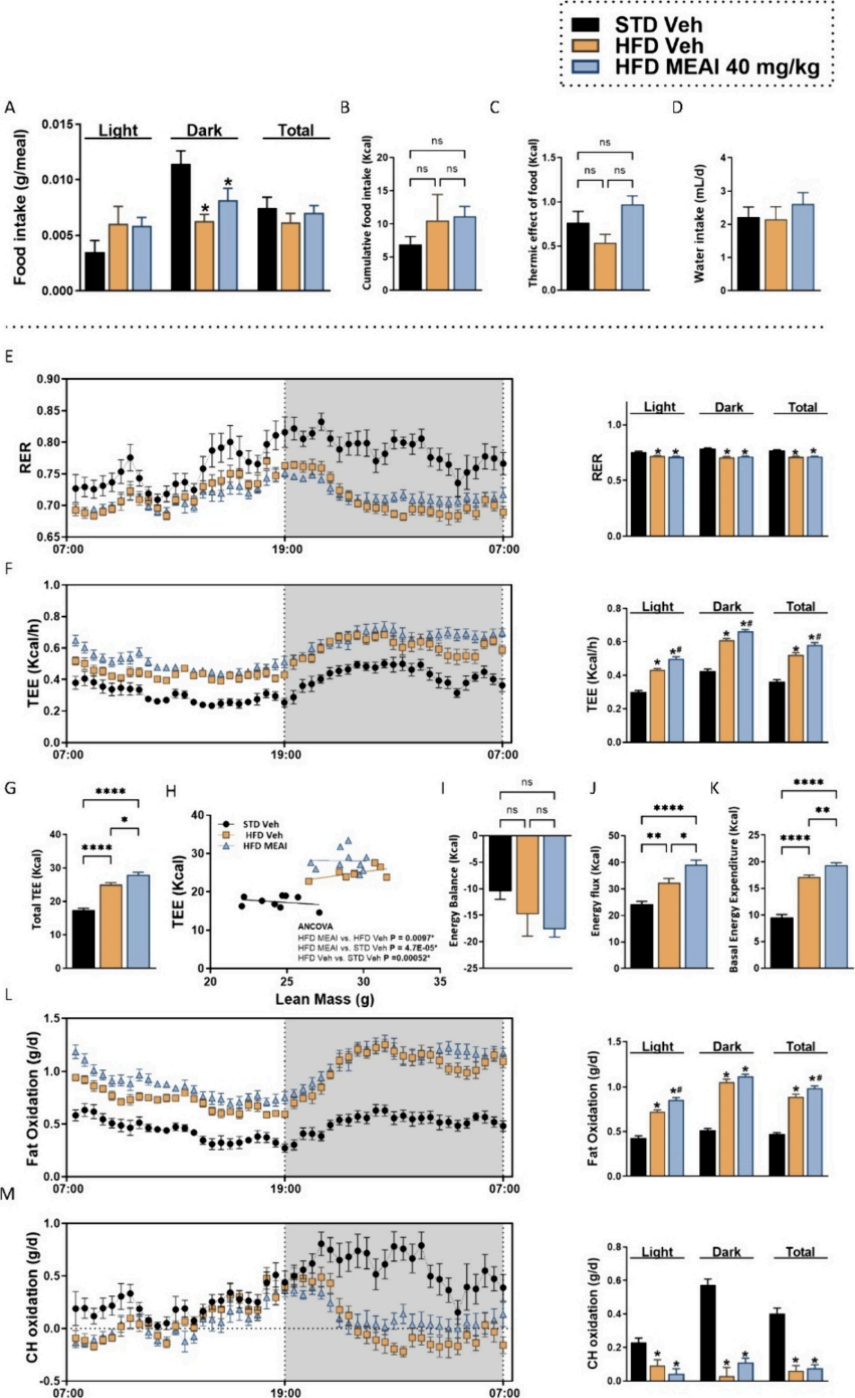
Chronic effects of MEAI treatment on food consumption and energy metabolism in DIO mice. Meal size (gram/uptake event) (A), cumulative food consumption (gram/day) (B), thermic effect of food (kcal) (C), sum of water intake (D), respiratory exchange rate (RER) (E), average energy expenditure rate (TEE) (F), cumulative energy expenditure (G), TEE of the three treatment groups was compared by ANCOVA using lean mass as covariant with the online tool provided by the NIDDK Mouse Metabolic Phenotyping Centers (MMPC, www.mmpc.org) (H), energy balance (I), energy flux (J), basal energy expenditure (K), fat oxidation (L), and carbohydrate oxidation (M). Data represent mean ± SEM from 8–11 mice per group. *P < 0.05 relative to STD vehicle; #*P* < 0.05 relative to HFD vehicle.

Mice consuming the HFD displayed altered feeding patterns, regardless of MEAI treatment. They exhibited a significant reduction in the meal size, as evidenced by a decrease in the food intake per meal (Figure 5A). This suggests a potential dampening of appetite in response to the HFD. However, this reduced meal size was counterbalanced by the inherently higher caloric density of the HFD, resulting in similar total food intake over a 24 h period across all groups (Figure 5B). Notably, the thermodynamic effect of food, which reflects the energy expended during digestion and absorption, also remained unchanged between groups (Figure 5C). Consistent with our previous observations in lean animals under acute conditions (Figure 1D), MEAI administration did not exert any significant influence on water consumption in DIO mice (Figure 5D).

Metabolically, the RER was slightly decreased in both the HFD vehicle and MEAI-treated groups in comparison to that of the STD vehicle group (Figure 5E). MEAI administration slightly increased the oxygen consumption and carbon dioxide production compared to the HFD vehicle-treated group (Figure S2A,B). Notably, the MEAI-treated group showed a significant increase in the average energy expenditure compared to both the HFD and STD vehicle-treated groups, with a clear elevation observed during both light and dark phases (Figure 5F), culminating in increases in cumulative TEE, suggesting a sustained effect on metabolism (Figure 5G). Importantly, ANCOVA revealed that these significant differences in TEE were treatment-dependent (*p* = 0.0097 vs HFD vehicle) (Figure 5H and Table S2). While no significant changes in overall energy balance were detected across the groups (Figure 5I), MEAI treatment did influence substrate utilization, demonstrating a significant increase in energy flux compared to vehicle groups (Figure 5J), suggesting heightened metabolic activity. In addition, MEAI significantly boosted basal energy expenditure (Figure 5K), potentially promoting calorie burning, even at rest. Furthermore, MEAI treatment led to an increase in the overall rate of FO compared with both HFD- and STD-vehicle groups (Figure 5L). This aligns with the observed decrease in RER and suggests that MEAI may promote the use of fat for energy. However, CHO levels were markedly reduced in both vehicle- and MEAI-treated HFD groups with no direct effect of the drug itself observed (Figure 5M). This finding warrants further investigation to understand the underlying mechanisms by which MEAI affects nutrient trafficking.

**Figure 6:**
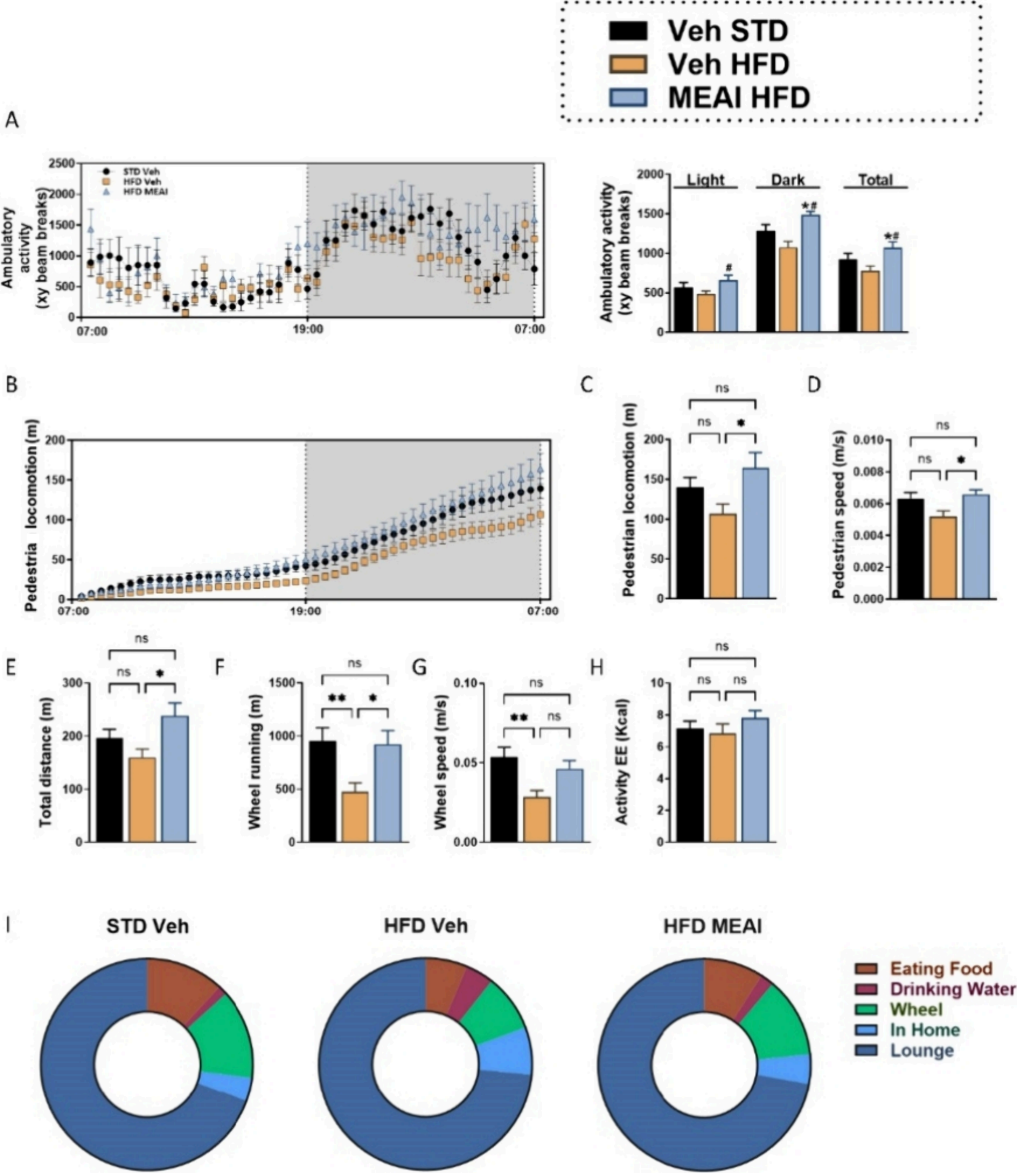
Locomotive activity is normalized following MEAI administration in an HFD-induced obesity model. Ambulatory activity (XY beam breaks) (A), pedestrian locomotion (B), sum of pedestrian distance (C), pedestrian locomotion speed (D), total distance (E), wheel running (F), wheel running speed (G), activity-related energy expenditure (H), and the time budget chart showing the average time spent on different activities within the home cage (I). Data represent mean ± SEM from 8–11 mice per group. *P < 0.05 relative to STD vehicle; #*P* < 0.05 relative to HFD vehicle.

Our analysis revealed that MEAI treatment significantly boosted voluntary ambulatory behaviors in DIO mice, including both pedestrian activity and grooming (Figure 6A). This effect was particularly pronounced during the nocturnal phase, aligning with the natural activity patterns. Notably, while MEAI treatment increased pedestrian activity, speed, and total distance traveled compared to the HFD-vehicle group (Figure 6B–E), it did not surpass the levels observed in the STD-vehicle group. This suggests that MEAI enhances activity without inducing an overstimulation.

A similar pattern emerged in the voluntary wheel running. MEAI-treated animals displayed an increased capability to run on the voluntary wheel, achieving speeds comparable to the STD-vehicle-treated group (Figure 5F,G). While the analysis of energy expenditure associated with activity levels showed a trend toward an increase in the MEAI group, this trend did not reach statistical significance (Figure 5H). Further analysis of cage activity revealed a clear preference for voluntary activities in MEAI-treated mice. They exhibited increased engagement with the running wheel, pedestrian locomotion, and extended interaction times with food and water dispensers (Figure 5I). This suggests that MEAI treatment not only enhances physical activity but also promotes a more active engagement with their environment.

### MEAI Improves Glycemic Control in DIO Mice

**Figure 7:**
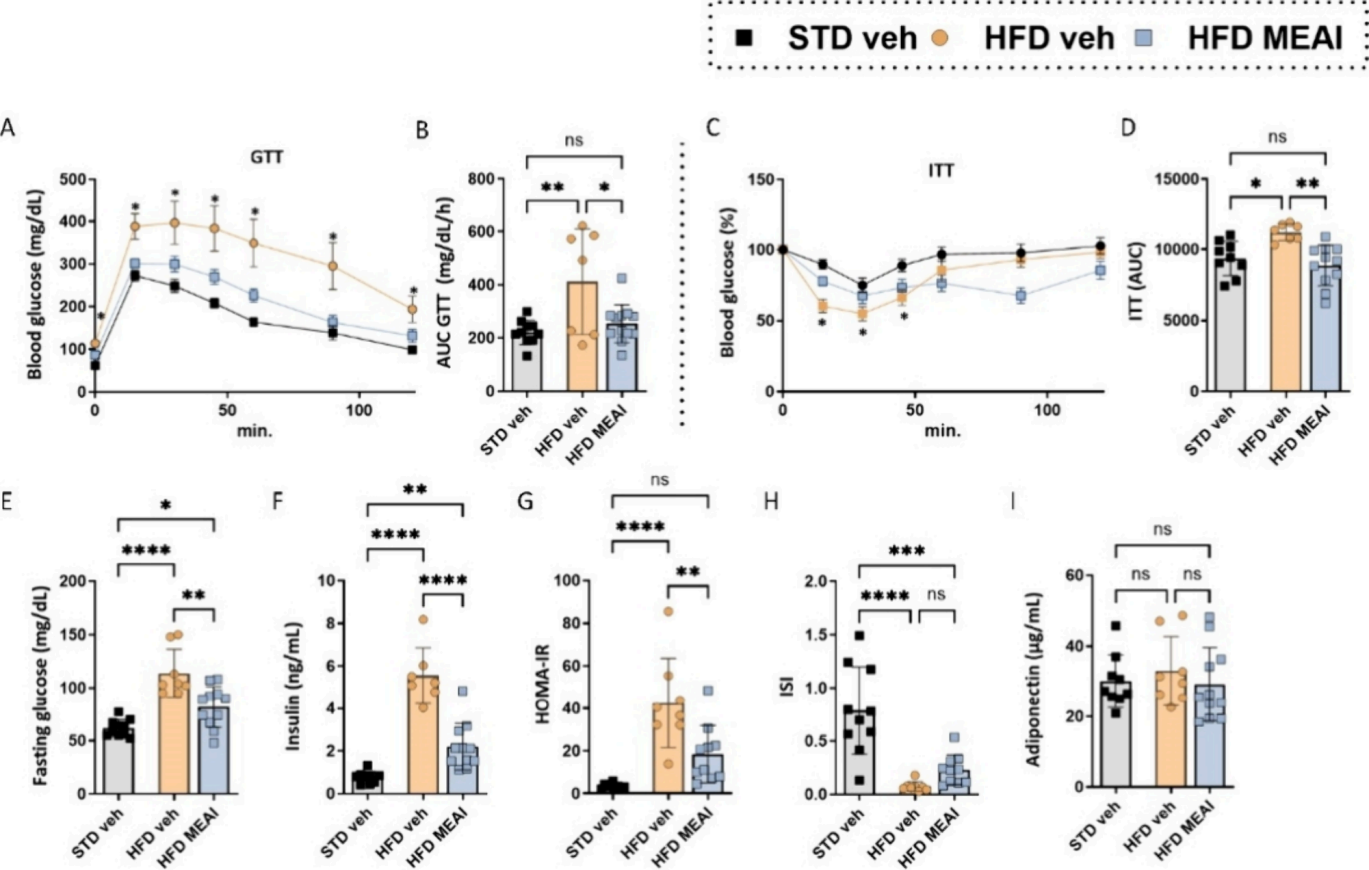
Effects of MEAI on glucose tolerance and insulin sensitivity. Glucose tolerance test (A), area under the (AUC) curve of the glucose tolerance test (B), insulin tolerance test (C), area under the curve (AUC) of insulin tolerance test (D), fasting blood glucose levels (E), serum insulin levels (F), HOMA-IR (G), ISI (H), and serum adiponectin levels (I). Data represent mean ± SEM from 8–11 mice per group. *P < 0.05 relative to STD-vehicle; #*P* < 0.05 relative to HFD vehicle.

Obesity is a well-known contributor to insulin resistance and hyperglycemia, which can ultimately lead to the onset of diabetes. In our DIO model, we observed a substantial impairment of glucose tolerance and an increase in hyperinsulinemia, as demonstrated by the results of glucose and insulin tolerance tests. However, following treatment with MEAI, we observed a significant improvement in glucose metabolism (Figure 7A–D), with fasting blood glucose and insulin levels also being reduced (Figure 7E,F). The beneficial effects of MEAI were further corroborated by improved HOMA-IR and ISI scores (Figure 7G,H), which are recognized markers of insulin resistance/sensitivity. These findings collectively suggest that MEAI exerts a positive influence on glucose homeostasis, potentially by improving the body’s capacity to utilize insulin and maintain healthy blood sugar levels. Interestingly, the circulating levels of adiponectin, an adipokine-related hormone known for its insulin-sensitizing properties, remained unchanged across all groups (Figure 7I). Further investigation is warranted to elucidate the specific mechanisms underlying the beneficial effects of MEAI on glucose metabolism, potentially including pathways independent of adiponectin.

### MEAI Ameliorates HFD-Induced Dyslipidemia

**Figure 8:**
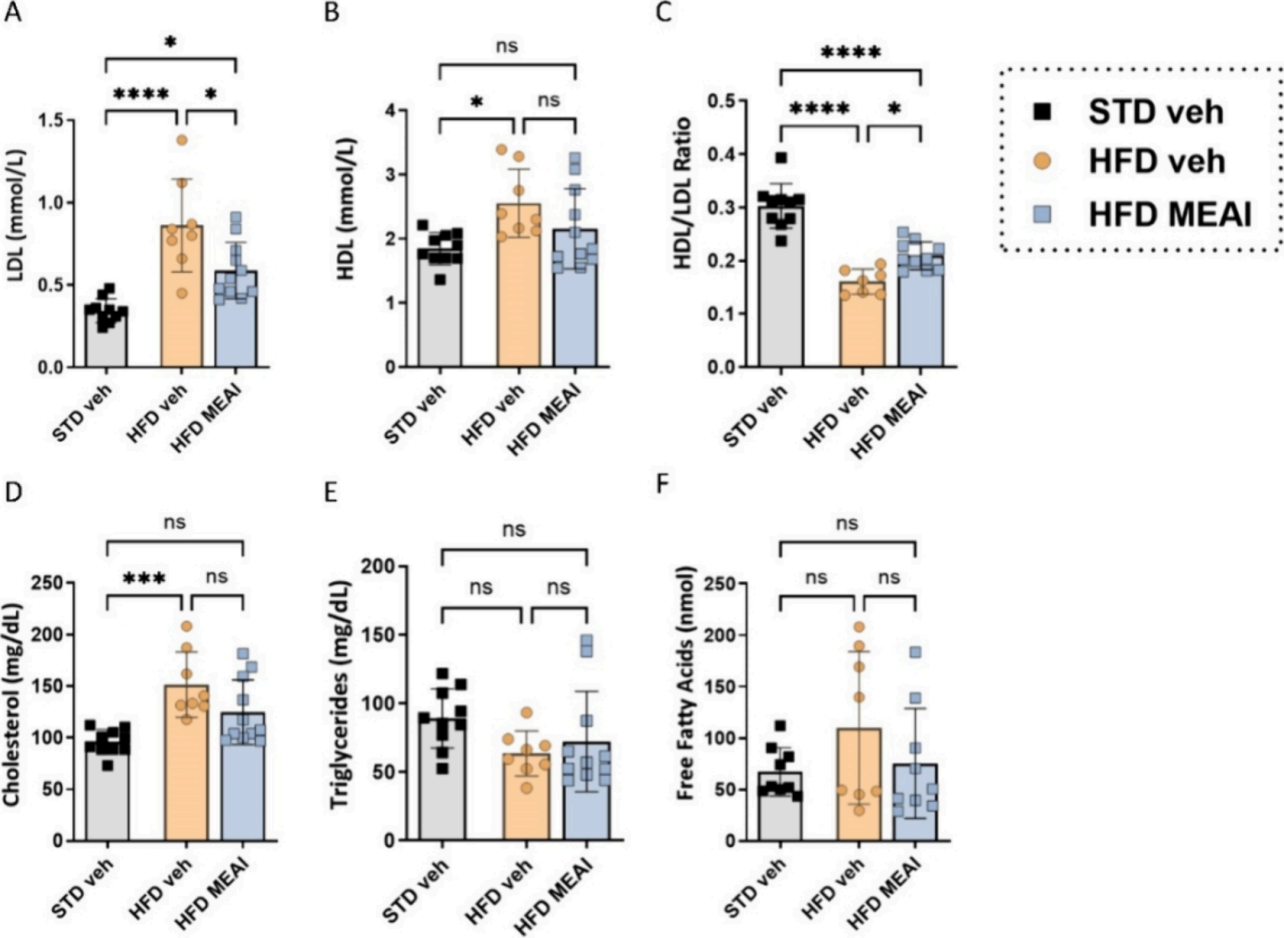
The profile of circulating lipids following MEAI treatment. LDL levels (A), HDL levels (B), HDL/LDL ratio (C), cholesterol levels (D), triglyceride levels (E), and free fatty acid serum levels (F). Data represent mean ± SEM from 8–11 mice per group. *P < 0.05 relative to STD vehicle; #*P* < 0.05 relative to HFD vehicle.

To investigate whether MEAI can alleviate dyslipidemia commonly associated with obesity, we analyzed the blood lipid profile. Our analysis revealed a significant decrease in LDL, a major risk factor for cardiovascular disease (CVD), in the MEAI-treated group compared to the HFD vehicle group (Figure 8A). This reduction is particularly noteworthy, because it occurred without a significant change in HDL levels (Figure 8B). HDL, often referred to as “good cholesterol”, plays a crucial role in removing cholesterol from the bloodstream. Consequently, the MEAI group exhibited a positive shift in the HDL-to-LDL ratio, a recognized marker of overall lipid health (Figure 8C). This finding suggests that MEAI treatment may exert beneficial effects on lipid metabolism, potentially contributing to improved cardiovascular health.

While total cholesterol levels displayed a downward trend in the MEAI-treated group (Figure 8D), this change did not reach statistical significance. Similarly, the circulating triglyceride levels remained largely unchanged across all groups (Figure 8E). However, a trend toward reduced free fatty acids, another potential contributor to CVD, was observed in the MEAI group (Figure 8F). Collectively, these results suggest that MEAI may offer therapeutic potential in improving the dyslipidemia associated with obesity. Further investigation is warranted to explore the underlying mechanisms by which MEAI exerts these effects and to determine the long-term efficacy of this treatment strategy.

### MEAI Reversed Obesity-Induced Hepatic Dysfunction and Steatosis

**Figure 9:**
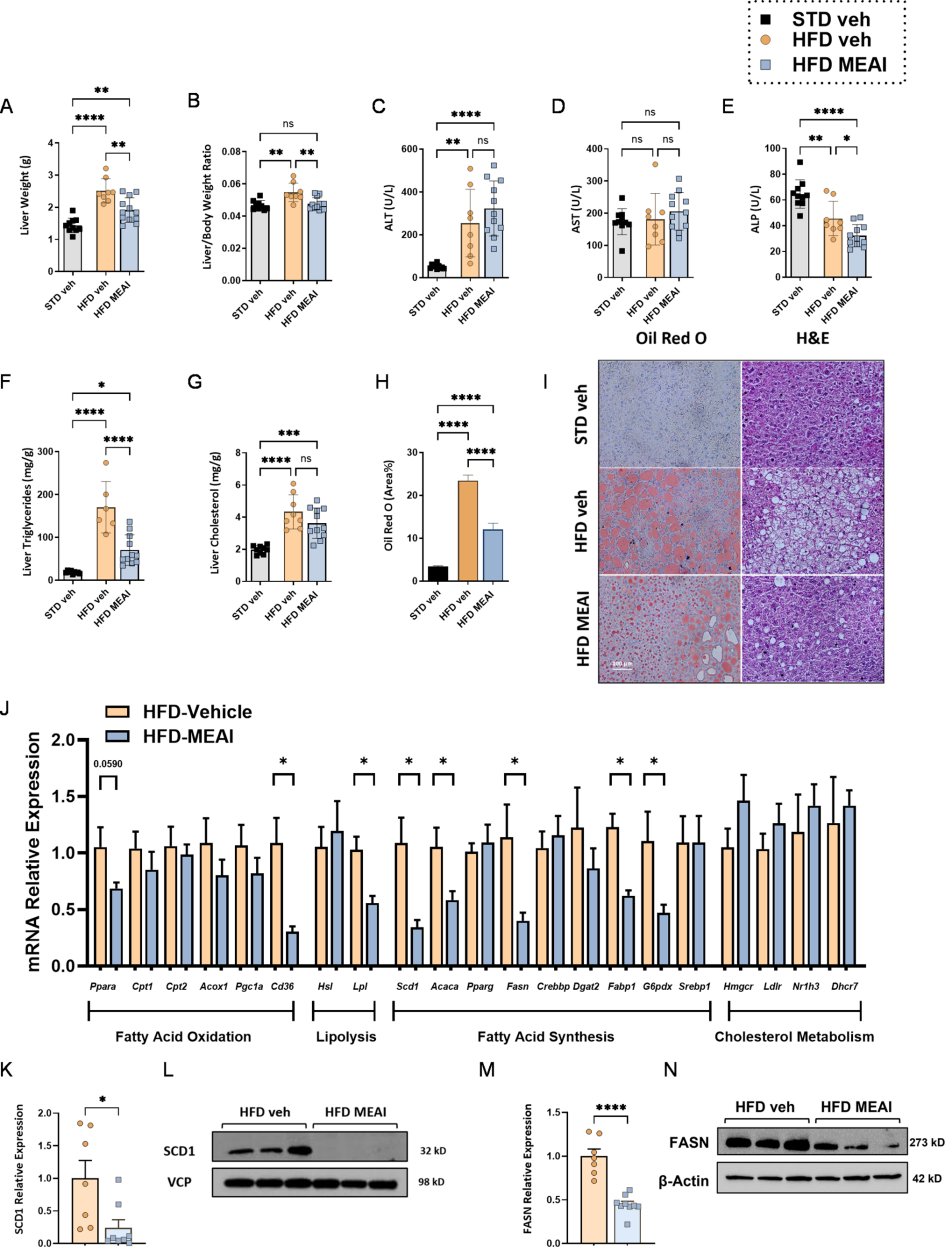
MEAI improves the obesity-associated liver steatosis. Liver weight (A), Liver weight to body weight ratio (B), liver enzyme levels ALT (C), AST (D), and ALP (E). Hepatic triglyceride content (F). Hepatic cholesterol content (G). Hepatic lipid content measured by Oil Red O staining (H).` Representative images of H&E as well as Oil Red O-stained specimen demonstrating lipid vacuoles in hepatocytes (I). Liver gene expression of fatty acids metabolism (J), and quantified hepatic protein levels measured by Western blotting of SCD1 (K, L) and FASN (M, N). Data represent mean ± SEM from 6–11 mice per group. *P < 0.05 relative to STD-vehicle; #*P* < 0.05 relative to HFD vehicle.

Obesity is a well-established risk factor for the development of MASLD, characterized by hepatic steatosis resulting from an imbalance between hepatic fatty acid uptake, synthesis, oxidation, and export.^22^ Given the promising effects of MEAI on body weight, FO, and circulating lipid levels, we then investigated its impact on liver steatosis. Our findings demonstrate that MEAI treatment reduced liver weight (Figure 9A) and normalized its ratio to body weight in HFD-fed mice (Figure 9B). Although MEAI administration did not significantly alter ALT or AST levels compared with the HFD-vehicle group, it significantly reduced ALP levels, which may suggest reduced liver injury (Figure 9C–E). Moreover, treatment with MEAI had a positive effect on hepatic lipid accumulation, as evidenced by the significant reductions in liver triglycerides compared to the HFD vehicle-treated controls and a trend toward a reduction in hepatic cholesterol levels (Figure 9F,G). These findings were further supported by a decrease in Oil Red O staining and reduced lipid vacuole numbers in MEAI-treated livers compared to the HFD vehicle-treated control group (Figure 9H,I). Overall, these findings suggest that MEAI may have a beneficial effect on hepatic lipid accumulation and liver function in the context of obesity-associated MASLD.

Following our observation of MEAI’s effect on hepatic fat accumulation, we next investigated its molecular mechanism by evaluating the mRNA expression of key genes involved in hepatic lipid metabolism using quantitative reverse transcriptase PCR (qRT-PCR) in DIO mice with or without MEAI administration (Figure 9J). This analysis focused on genes regulating various aspects of fatty acid metabolism, including those responsible for fatty acid oxidation (FAO). We examined genes like peroxisome proliferator-activated receptor α (*Ppara*), carnitine palmitoyl transferase 1 and 2 (*Cpt1* and *Cpt2*), acyl-CoA oxidase 1 (*Acox1*), PPARγ coactivator 1 alpha (*Pgc1a*), and the fatty acid transporter *Cd36*. MEAI treatment significantly downregulated the mRNA expression of *Cd36* (*p* < 0.05), suggesting reduced fatty acid uptake by hepatocytes. This finding aligns with the observed decrease in fat accumulation and implies a potential mechanism by which MEAI exerts its beneficial effects. Interestingly, protein levels of CD36 remained unchanged (Figure S3A), indicating a possible post-transcriptional regulatory mechanism or a time-dependent effect of MEAI on protein expression. Further investigation is warranted to explore this discrepancy.

We further assessed the expression of genes that regulate fatty acid lipolysis. MEAI treatment resulted in a significant decrease in the mRNA level of lipoprotein lipase (*Lpl*) (*p* < 0.05). However, no significant changes were observed in the mRNA expression of hormone-sensitive lipase (*Hsl*). These findings suggest that MEAI may primarily target LPL-mediated lipolysis, potentially contributing to the observed reduction in fat accumulation. The most prominent effects of MEAI were observed in genes that regulate fatty acid synthesis. MEAI significantly decreased the mRNA expression of key lipogenic enzymes, including stearoyl-CoA desaturase 1 (*Scd1*), acetyl-CoA carboxylase alpha (*Acaca*), fatty acid synthase (*Fasn*), fatty acid-binding protein 1 (*Fabp1*), and glucose-6-phosphate dehydrogenase (*G6pdx*). Conversely, MEAI did not affect the expression of PPAR gamma (*Pparg*), CREB-binding protein (*Crebp*), diacylglycerol acyltransferase 2 (*Dgat2*), or sterol regulatory element-binding protein 1 (*Srebp1*). Evaluation of genes associated with cholesterol metabolism, including HMG-CoA reductase (*Hmgcr*), LDL receptor (*Lldr*), liver X receptor alpha (*Nr1h3*), and 7-dehydrocholesterol reductase (*Dhcr*), revealed no significant changes upon MEAI treatment. This suggests that MEAI’s hepatic effects are primarily focused on fatty acid metabolism. To corroborate the observed changes in mRNA expression, we further assessed the protein levels of key regulators involved in fatty acid metabolism. Consistent with the mRNA data, MEAI treatment significantly reduced the protein levels of SCD1 (Figure 9K,L) and FASN (Figure 9M,N), further substantiating its inhibitory effect on lipogenesis. Additionally, MEAI displayed a trend toward increased phosphorylated AMPK (p-AMPK) (Figure S3B), a known activator of FAO. Conversely, MEAI did not significantly affect the levels of phosphorylated ACC (p-ACC) (Figure S3C). The observed increase in p-AMPK, coupled with unchanged p-ACC, suggests a potential activation of the AMPK signaling pathway by MEAI, which may contribute to the reduction in lipogenesis. Taken together, these results demonstrate that MEAI exerts its antisteatotic effect by suppressing fatty acid uptake and lipogenesis, offering a promising therapeutic strategy for MASLD.

## Discussion

Obesity presents a complex therapeutic challenge, as currently available pharmaceutical and lifestyle-based interventions often fail to provide sustained and healthy weight loss and have side effects requiring long-term treatment regimens.^23,24^ Despite recent progress in antiobesity drug development, the need for effective and beneficial therapies remains urgent. The reemergence of interest in psychedelic compounds offers promising prospects for treating various recalcitrant behavioral and neuropsychiatric disorders,^25^ although their potential for treating obesity has yet to be fully explored. Our study provides the first preclinical evidence for the efficacy of the novel psychoactive substance, MEAI, in regulating energy metabolism and mitigating obesity and its related metabolic abnormalities. MEAI demonstrated an effect in alleviating various conditions associated with metabolic syndrome in a DIO mouse model. In addition to mitigating adiposity and reducing body weight, MEAI also improved glucose homeostasis, lowered dyslipidemia, and preserved liver function, possibly by improving fat utilization and restricting *de novo* hepatic lipogenesis. These findings suggest that MEAI may have potential as a novel therapeutic option for obesity and related metabolic disorders, warranting further investigation in preclinical and clinical settings.

Clinical research has been exploring the potential of classic psychedelics, including LSD, 3,4-methylenedioxymethamphetamine (MDMA), and psilocybin, for treating a range of neuropsychiatric disorders including addiction, depression, post-traumatic stress disorder, and anxiety. Encouraging findings have emerged from past and ongoing trials, highlighting the potential therapeutic utility of these compounds.^26−28^ In a recent open-label feasibility study, 10 adult female participants with anorexia nervosa (AN), a common eating disorder, received a single 25 mg dose of synthetic psilocybin alongside psychological support. The treatment demonstrated safety, tolerability, and acceptability, with no clinically significant adverse events observed; these results are promising considering the lack of proven treatments for AN.^29^ On the other hand, the potential of psychedelic compounds for treating binge eating and obesity remains largely unexplored, with current research on this topic being limited and inconclusive.^30^

The central serotonergic system has been identified as a promising target for developing drugs that combat obesity due to the essential role of the neurotransmitter serotonin in appetite and satiety regulation. However, drugs that increase serotonin (5-HT) levels by either direct release or reuptake inhibition have been associated with severe side effects, leading to their withdrawal from the market.^12,31^ To minimize off-target effects, researchers have focused on developing receptor-specific ligands. A specific 5-HT2C agonist, lorcaserin, demonstrated promising antiobesity effects but was withdrawn due to cancer concerns.^32^ Similarly, preclinical studies have identified other receptor subtypes, such as 5-HT1B, 5-HT2B, and 5-HT6, as potential targets for developing antiobesity medications.^33,34^ While the precise mechanism of action of MEAI remains unclear, Shimshoni and colleagues investigated its potential impact on key monoaminergic targets, including the monoamine metabolizing enzymes (MAO) A and B and the vesicular monoamine transporter (VMAT). The results of radioligand displacement assays indicated that MEAI exhibited modest binding inhibition to the 5-HT2B receptor, while the other targets were unaffected.^35^ Furthermore, the findings suggest that MEAI’s lack of inhibitory activity on MAO-A, MAO-B, and VMAT may mitigate the risk of cardiovascular crisis and serotonin syndrome, which are associated with 5-HT2B activation.

Halberstadt et al. conducted *in vitro* binding studies on MEAI, investigating its interactions with various CNS receptors and transporters. The results showed that MEAI interacts with plasma membrane dopamine transporter (DAT), norepinephrine transporter (NET), and serotonin transporter (SERT), along with serotonergic 5-HT1A and 5-HT2B receptors and α2-adrenergic receptors. Notably, MEAI displayed moderate affinities for the 5-HT1A and 5-HT2B receptors (*K*_*i*_ of 2503 and 4793 nM, respectively) while showing higher affinities for the α2 subtypes (*K*_*i*_ of 751 to 1555 nM).^36^ To gain further insight into its mechanism of action, we screened MEAI against 87 receptors, enzymes, and transporters at a concentration of 10 μM. Notably, inhibitory activity above 50% was observed with several serotonergic receptors (5-HT1A, 5-HT2A, and 5-HT2B), with the dopamine D2 receptor, and conversely with the MAO-A enzyme (Figure S4A). Considering the safety concerns linked to 5-HT2B activation, we evaluated MEAI’s functionality as a calcium flux agonist on this receptor, revealing that MEAI did not act as an agonist at the 5-HT2B receptor, as evidenced by its maximum response of 18.644% compared to serotonin’s positive control response of 99.891% enzyme (Figure S4B,C).

The regulation of energy homeostasis, the balance between energy intake and expenditure, is mediated by anabolic and catabolic pathways.^37^ The development of obesity is caused by a prolonged energy imbalance, where energy intake exceeds expenditure. Mitochondrial dysfunction, particularly in lipid substrate oxidation, can also lead to energy expenditure imbalances and metabolic diseases.^38^ Conversely, effective management of FAO can help resist DIO.^39^ Interestingly, our investigation on MEAI revealed that acute treatment in lean mice and chronic dosing in obese animals did not affect food intake but significantly increased TEE, REE, and FO. This suggests that MEAI may modulate energy balance and promote weight loss independently of food intake. In line with earlier findings, the safety and tolerability of MEAI were confirmed at doses of 40 and 60 mg/kg, although caution should be exercised when administering higher doses of up to 100 mg/kg, as some adverse effects were observed.^17^ Our findings also indicated a decrease in sucrose preference after the acute intake of MEAI, suggesting an interference with the reward circuitry associated with palatable foods, thus indicating that MEAI could potentially be utilized to treat binge/compulsive eating behavior. These results are consistent with previous studies on psilocybin, a serotonergic psychedelic compound.^40^

Rodent models are valuable for studying human metabolism, as they can replicate physiological responses to DIO and resistance to weight loss through compensatory mechanisms. These models are also useful in predicting the efficacy of potential antiobesity drugs.^41−43^ However, preclinical studies investigating the effects of psychedelics on murine models of DIO are limited. Huang et al. reported that psilocybin reduced weight gain in rats fed a cafeteria diet but had no effect on weight loss at the given doses.^44^ Likewise, Fadahunsi et al. found that psilocybin had no impact on body weight or food intake in various conditions, including lean mice, DIO mice, and genetic models of obesity (*ob*/*ob* mice and melanocortin-receptor 4 knockout mice).^40^ In contrast, our study is the first to demonstrate that MEAI significantly induced weight loss and beneficial changes in body composition in DIO mice without altering food intake. Interestingly, the interaction time with the food dispenser was significantly shorter in the MEAI-treated group, further suggesting that MEAI may mitigate binge-eating behaviors. Furthermore, MEAI normalized the voluntary behavioral and activity patterns of obese mice, indicating its potential to reverse the sedentary behavior associated with obesity.^41^

Several studies have established a close association between central adiposity, prediabetes, insulin resistance, and liver function, making obesity a significant risk factor for T2D and MASLD.^22,45,46^ In the DIO model, MEAI demonstrated promising abilities to preserve glucose homeostasis by enhancing glucose tolerance and attenuating insulin resistance, reducing dyslipidemia, and enhancing liver health through reduced hepatic lipid accumulation. Remarkably, the 5-HT2B receptor has demonstrated intriguing dual effects on glucose homeostasis and liver function. On the one hand, 5-HT2B agonists have been shown to promote insulin secretion, partly regulated by an increase in intracellular Ca^2+^ and enhanced mitochondrial activity.^47^ Additionally, increased expression of 5-HT2B in mouse islets during pregnancy has been shown to enhance β cell proliferation while its pharmacological blockade impaired glucose tolerance, suggesting that this receptor could be a favorable target for treating gestational diabetes and T2D.^48^

Sumara et al. revealed that fasting-induced 5-HT2B receptor signaling through gut-derived serotonin (GDS) promotes gluconeogenesis and hinders hepatic glucose uptake, involving a glucose transporter 2-dependent mechanism.^49^ Conversely, contrasting findings indicate that 5-HT enhances hepatic glucose uptake and intrahepatic fat content in dogs,^50^ while in *ob*/*ob* mice, increased duodenal 5-HT content can be alleviated with a 5-HT3 receptor antagonist, which boosts the SERT activity in the duodenum.^51^ Moreover, activation of 5-HT2B has been linked to a detrimental impact on MASLD progression, involving mTOR activation and subsequent increased hepatic triglyceride production and circulating free fatty acids.^52^ Encouragingly, a 5-HT2B antagonist demonstrated efficacy in attenuating hepatic fibrosis and improving hepatic function in a murine chronic liver inflammation model.^53^ The divergent findings concerning 5-HT2B signaling emphasize its intricate and multifaceted role in metabolic and hepatic regulation, warranting additional research to gain a comprehensive understanding of its action in these disorders. Currently, it remains uncertain whether the effects of MEAI on the liver are solely linked to 5-HT2B receptor modulation or whether an alternative mechanism is at play, underscoring the imperative for further investigation.

In our study, we also shed light on the molecular mechanisms underlying the beneficial effects of MEAI on hepatic lipid metabolism in the context of obesity-associated MASLD. Our investigation revealed significant downregulation of *Cd36* mRNA expression upon MEAI treatment, suggesting a reduction in fatty acid uptake by hepatocytes and providing mechanistic insight into the lipid-lowering effects of MEAI. Interestingly, while mRNA levels of *Cd36* were affected, protein levels remained unchanged, indicating the presence of potential post-transcriptional regulatory mechanisms or temporal dynamics in protein expression that warrant further exploration.^54^ Furthermore, MEAI treatment led to a significant decrease in the levels of mRNA expression of key lipogenic enzymes, including *Scd1*, *Acaca*, *Fasn*, *Fabp1*, and *G6pdx*, highlighting its inhibitory effect on *de novo* hepatic lipogenesis. These findings were further supported by the corresponding reduction in protein levels of SCD1 and FASN.^55,56^ Additionally, MEAI treatment displayed a trend toward increased p-AMPK, suggesting potential activation of the AMPK signaling pathway, which may contribute to the suppression of lipogenesis.^57^ Overall, our study provides compelling evidence elucidating the molecular mechanisms underlying the antisteatotic effects of MEAI, offering promising avenues for future research and clinical translation in the management of MASLD.

In conclusion, this study provides novel insights into the effects of psychedelics on energy balance and obesity. Our results demonstrate that administration of MEAI, both acutely and chronically, leads to significant changes in energy homeostasis, resulting in a reduction of obesity and its associated complications. While the precise mechanisms by which MEAI exerts these effects are not fully elucidated, its specificity for certain receptors and lack of activation at the 5-HT2B receptor show promise in minimizing undesirable side effects. Thus, our findings strongly suggest that further investigation into MEAI as a possible therapeutic option for obesity and its metabolic comorbidities is warranted.

While our study provides valuable insights into the therapeutic potential of MEAI in mitigating obesity and its related metabolic abnormalities in male mice, it is important to note that we did not include female mice in our experimental design. Given the emerging interest in understanding the impact of sex on drug efficacy and safety,^58^ future studies will aim to incorporate both male and female animals to further evaluate the therapeutic potential of MEAI in addressing obesity and its metabolic sequelae.

## Supporting Information Available

(Figure S1) Effects of acute MEAI administration on respiratory parameters; (Figure S2) effects of chronic MEAI administration on respiratory parameters in diet-induced obesity model; (Figure S3) effects of MEAI administration on protein levels of lipid metabolism regulators; (Figure S4) MEAI binding affinity to serotonin receptors and calcium influx agonist screening; (Table S1) real-time PCR primer sequences; and (Table S2) energy expenditure adjusted for lean mass using ANCOVA (PDF)
