# Supplementary material for: 5-Methoxy-2-aminoindane Reverses Diet-Induced Obesity and Improves Metabolic Parameters in Mice: A Potential New Class of Antiobesity Therapeutics
Source: ACS Pharmacol Transl Sci. 2024 Jul 30;7(8):2527–43. doi: 10.1021/acsptsci.4c00353 (PMC11320730; doi:10.1021/acsptsci.4c00353)
Supplement: Supplementary file 1 — pt4c00353_si_001.pdf [file pt4c00353_si_001.pdf]

Supporting Information

5-methoxy-2-aminoindane Reverses Diet-Induced Obesity and Improves Metabolic Parameters in Mice: A Potential New Class of Anti-Obesity Therapeutics

Saja Baraghithy[1], Asaad Gammal[1], Anna Permyakova[1], Sharleen Hamad[1], Radka Kocvarova[1], Yael Calles[1], and Joseph Tam[1,*]

[1]Obesity and Metabolism Laboratory, The Institute for Drug Research, School of Pharmacy, Faculty of Medicine, The Hebrew University of Jerusalem, Jerusalem 9112001, Israel

***Correspondence:** Joseph Tam; E-mail: yossi.tam@mail.huji.ac.il

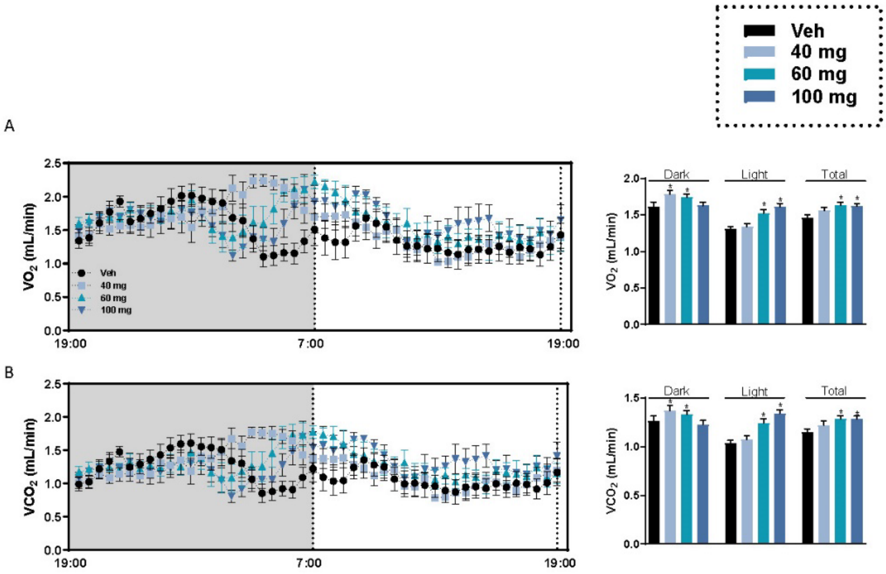

Figure S1: Effects of acute MEAI administration on respiratory parameters. Rate of oxygen consumption (VO_2) **A**, and Rate of carbon dioxide emission (VCO_2) **B**. Data represent the mean ± SEM from 6 to 8 mice per group. *P < 0.05 relative to Vehicle-treated group.

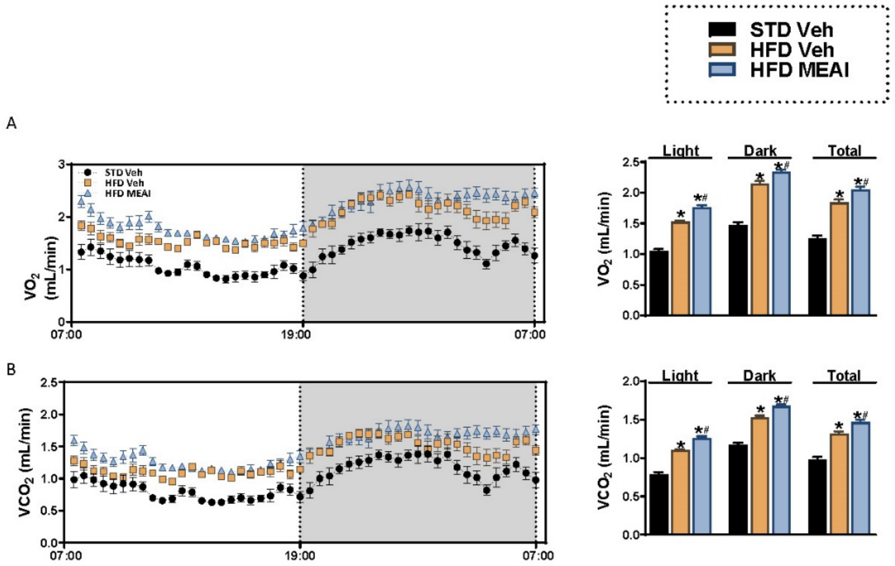

Figure S2: Effects of chronic MEAI administration on respiratory parameters in diet-induced obesity model. Rate of oxygen consumption (VO_2) **A**, and Rate of carbon dioxide emission (VCO_2) **B**. Data represent mean ± SEM from 8–11 mice per group. $*P < 0.05$ relative to STD-vehicle; $\#P < 0.05$ relative to HFD-vehicle.

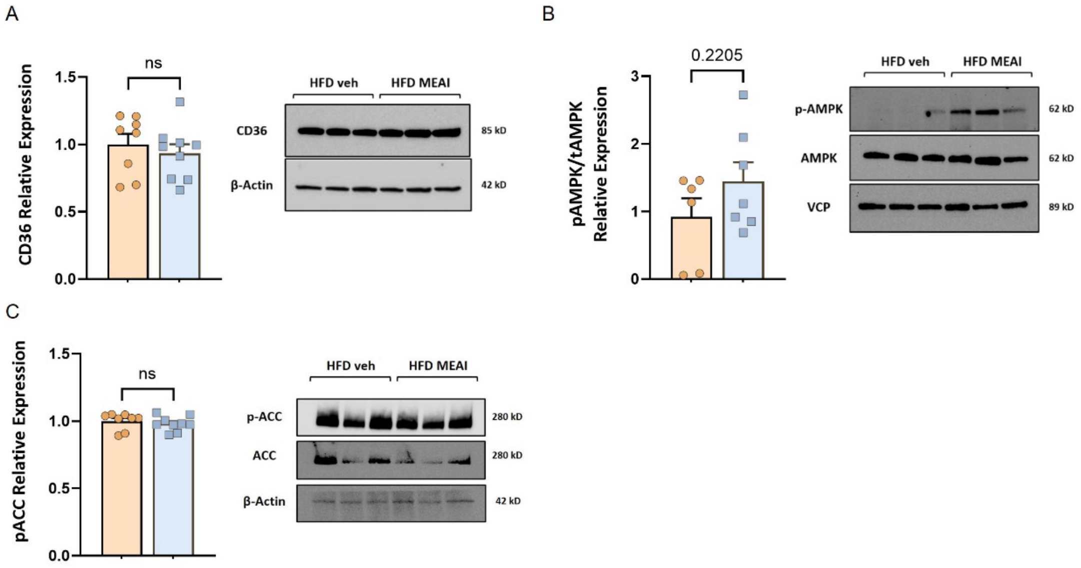

Figure S3: Effects of MEAI administration protein levels of lipid metabolism regulators. Quantification of protein levels of CD36 A, phosphorylated AMPK **B**, and phosphorylated ACC **C**. Data represent mean ± SEM from 6–9 mice per group. *P < 0.05 relative to HFD-vehicle.

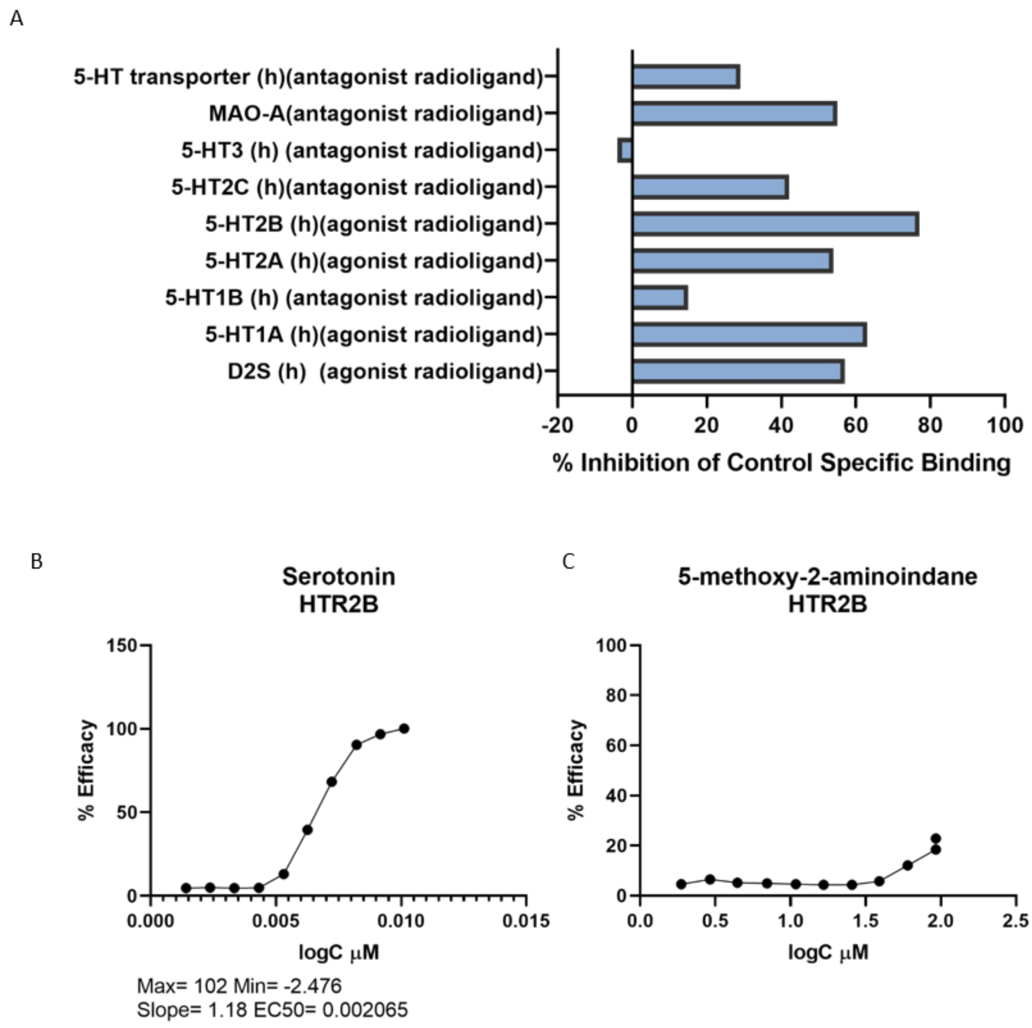

Figure S4: MEAI binding affinity to serotonin receptors and calcium influx agonist screening. Binding affinities of MEAI to several serotonin and serotonin-related receptors **A**, Calcium influx via HTR2B following Serotonin administration **B**, and Calcium influx via HTR2B following MEAI administration **C**.

Table S1. Real-Time PCR Primer Sequences

Gene	Forward	Reverse
Mus musculus *Ppara*	AGAGCCCCATCTGTCCTCTC	ACTGGTAGTCTGCAAAACCAAA
Mus musculus *Cpt1*	CCGTGAGGAACTCAAACCTATT	CAGGGATGCGGGAAGTATTG
Mus musculus *Cpt2*	CAGCACAGCATCGTACCCA	TCCCAATGCCGTTCTCAAAAT
Mus musculus *Acox1*	TAACTTCCTCACTCGAAGCCA	AGTTCCATGACCCATCTCTGTC
Mus musculus *Pgc1a*	AACCACACCCACAGGATCAGA	TCTTCGCTTTATTGCTCCATGA
Mus musculus *Cd36*	CCTCTGACATTTGCAGGTCTATC	GCATTGGCTGGAAGAACAAATC
Mus musculus *Hsl*	CCGCTGACTTCCTGCAAGAG	CTGGGTCTATGGCGAATCGG
Mus musculus *Lpl*	GGGAGTTTGGCTCCAGAGTTT	TGTGTCTTCAGGGGTCCTTAG
Mus musculus *Scd1*	TTCTTGCGATACACTCTGGTGC	CGGGATTGAATGTTCTTGTCGT
Mus musculus *Acaca*	TCTCCTGGTGACAATGCTTATT	GGGTTAGCTCTGTGAGGATATTT
Mus musculus *Pparγ*	CTGCTCAAGTATGGTGTCCATGA	TGAGATGAGGACTCCATCTTTATTCA
Mus musculus *Fasn*	CCTGACCAAGGTGCTGTTAT	CATCAAGAAGTGCTGGGATCT
Mus musculus *Crebp*	CATTGCCCCTGGAGTTGTTATG	TTCTCTTGCTGCCTCCCTGTT
Mus musculus *Dgat2*	GTGCCATCGTCTGCAAGATTC	GCATCACCACACACCAATTCAG
Mus musculus *Fabp1*	ATGAACTTCTCCGGCAAGTACC	CTGACACCCCCTTGATGTCC
Mus musculus *G6pdx*	CACAGTGGACGACATCCGAAA	AGCTACATAGGAATTACGGGCAA
Mus musculus *Srebp1*	GGAGCCATGGATTGCACATT	GCTTCCAGAGAGGAGGCCAG
Mus musculus *Hmgcr*	AGCTTGCCCGAATTGTATGTG	TCTGTTGTGAACCATGTGACTTC
Mus musculus *Ldlr*	TCCCTGGGAACAACTTCACC	CACTCTTGTCGAAGCAGTCAG
Mus musculus *Nr1h3*	CTCAATGCCTGATGTTTCTCCT	TCCAACCCTATCCCTAAAGCAA
Mus musculus *Dhcr*	AGGCTGGATCTCAAGGACAAT	GCCAGACTAGCATGGCCTG
Mus musculus *Ubc*	GCCCAGTGTTACCACCAAGA	CCCATCACACCCAAGAACA

Table S2. Energy expenditure adjusted for lean mass using ANCOVA.			
HFD-MEAI vs. HFD-Vehicle			
Variables	*Estimate*	*StdError*	*Pvalue*
(Intercept)	*19.57*	*11.74*	*0.1164*
Lean Body Mass	*0.2966*	*0.4069*	*0.4772*
Treatment	*-3.396*	*1.148*	***0.009791***
HFD-MEAI vs. STD-Vehicle			
Variables	*Estimate*	*StdError*	*Pvalue*
(Intercept)	*33.05*	*11.44*	*0.01065*
Lean Body Mass	*-0.1715*	*0.3962*	*0.6708*
Treatment	*-11.47*	*2.081*	***0.00004707***
HFD-Vehicle vs. STD-Vehicle			
Variables	*Estimate*	*StdError*	*Pvalue*
(Intercept)	*21.02*	*7.496*	*0.01405*
Lean Body Mass	*0.1321*	*0.2535*	*0.6104*
Treatment	*-6.825*	*1.524*	***0.0005191***
